# Reversible Suppression of Fear Memory Recall by Transient Circadian Arrhythmia

**DOI:** 10.3389/fnint.2022.900620

**Published:** 2022-05-27

**Authors:** Athreya Steiger, Julia Farfan, Nathan Fisher, H. Craig Heller, Fabian-Xosé Fernandez, Norman F. Ruby

**Affiliations:** ^1^Department of Biology, Stanford University, Stanford, CA, United States; ^2^Department of Psychology, University of Arizona College of Science, Tucson, AZ, United States

**Keywords:** freezing, siberian, hamster, suprachiasmatic, hippocampus, context, cue

## Abstract

We tested the hypothesis that a temporary period of circadian arrhythmia would transiently impair recall of an aversive memory in Siberian hamsters (*Phodopus sungorus*). Unlike mice or rats, circadian arrhythmia is easily induced in this species by a one-time manipulation of their ambient lighting [i.e., the disruptive phase shift (DPS) protocol]. Hamsters were conditioned to associate footshocks with a shock chamber (context) and with a predictive auditory tone (cue), and then exposed to the DPS protocol. Following DPS, animals either became arrhythmic (ARR), reentrained to the light-dark cycle (ENT), or became arrhythmic for < 14 days before their circadian locomotor rhythms spontaneously recovered and reentrained (ARR-ENT). Tests for contextual memory showed that freezing was decreased 9–10 days post-DPS when both ARR and ARR-ENT groups were arrhythmic. Once ARR-ENT animals reentrained (day 41), however, freezing was elevated back to Pre-DPS levels and did not differ from those observed in ENT hamsters. ENT animals maintained high levels of freezing at both time points, whereas, freezing remained low in ARR hamsters. In contrast to contextual responses, cued responses were unaffected by circadian arrhythmia; all three groups exhibited elevated levels of freezing in response to the tones. The differential impact of circadian arrhythmia on contextual versus cued associative memory suggests that arrhythmia preferentially impacts memory processes that depend on the hippocampus.

## Introduction

Siberian hamsters are uniquely suited for studies investigating the impact of circadian disruption on memory because their circadian timing can be eliminated easily by a simple one-time manipulation of their ambient lighting. This manipulation, termed the disruptive phase shift (DPS) protocol, eliminates circadian timing at the molecular level in the central circadian pacemaker within the hypothalamic suprachiasmatic nucleus (SCN; Grone et al., 2011). The main advantage of this model is that it induces arrhythmia under a light-dark cycle while leaving animals genetically and neurologically intact. In tests of object recognition, circadian-arrhythmic hamsters failed to recognize familiar objects only 20 min later, but not when tested 1 min after exploring them (Ruby et al., 2008). Thus, arrhythmia appears to impair long-term, but not short-term object recognition memory. When allowed to freely explore a T-maze to assess spatial working memory, circadian-arrhythmic animals fail to alternate, but instead, make random arm choices (Ruby et al., 2013; Fernandez et al., 2014). In both memory tasks, circadian-arrhythmic animals explore as much as circadian-entrained controls, which suggests that performance differences are due to cognitive rather than to motivational deficits (Ruby, 2021).

These observations led us to investigate the hippocampal circuits that underlie the memory processes involved in these tasks. We first examined brain oscillations in the theta range (5–8 Hz) because theta oscillations, as well as the cholinergic signals they initiate, are critically involved in both memory encoding for objects and spontaneous alternation behavior. Circadian-arrhythmia in Siberian hamsters shortened the duration of individual bouts of theta by ∼50%, but had no effect on the total time spent exploring objects or on the duration of individual bouts of object exploration (Loewke et al., 2020). In addition to fragmented theta signaling, we found that circadian arrhythmia was associated with a doubling of spontaneous inhibitory events recorded in the granule cells of the hippocampal dentate gyrus (McMartin et al., 2021). Dentate circuits in arrhythmic hamsters were also more resistant to cholinergic suppression of inhibitory signals by the cholinergic agonist carbachol (McMartin et al., 2021). These findings suggest that circadian-arrhythmia in the SCN impairs memory formation by interfering with cholinergic signaling and by increasing inhibition in dentate circuits.

While our past experiments have focused on spatial and recognition memory, the present study investigated whether circadian arrhythmia could impair recall of an aversive event. In past studies, induction of circadian arrhythmia by the DPS protocol remained stable for an indefinite period of time (Ruby et al., 1996). During our initial research with fear conditioning (FC), however, we found many animals in which the conditioning procedure prevented permanent arrhythmia. These animals became circadian-arrhythmic after the DPS treatment, but then spontaneously recovered entrained locomotor rhythms within the following 14 days. While we do not have an immediate explanation for this phenomenon, we were able to use it to test whether circadian arrhythmia could suppress memory recall in a reversible manner.

We also report an often disregarded measure of fear which quantifies the duration of individual bouts of freezing. Once an episode of freezing is initiated, it must also be terminated before volitional activity can resume. Termination of freezing may indicate a return to safety, but in the wild, it might also be followed by a different defense strategy such as fleeing or by patrolling immediate surroundings to check for predators (Blanchard et al., 1990; Eilam, 2005). The laboratory apparatus for testing fear in response to footshocks does not allow animals to engage in either fleeing or patrolling, thus, this constraint may result in other manifestations of fear, such as an increased reluctance to resume locomotion. We therefore hypothesized that increased fear might prolong individual episodes of freezing rather than increasing the number of freezing episodes.

## Materials and Methods

### Animals and Housing Conditions

Siberian hamsters (*Phodopus sungorus*) were bred in the laboratory in a 16:8-h light-dark (LD) cycle (lights on at 0200 h, PST) at an ambient temperature of 22°C. Animals were provided with natural cotton batting for nesting material; food (Purina chow #5015) and tap water were available *ad libitum*. All experimental procedures were approved by Stanford University’s Administrative Panel on Laboratory Animal Care (Animal Use Protocol #14988) and were conducted in accordance with the NIH Guide for the Care and Use of Laboratory Animals. Housing and lighting conditions were as described previously (Ruby et al., 2010; Fernandez et al., 2014). Prior to the start of an experiment, animals were housed individually for 14 days. Locomotor activity was measured by passive infrared motion detectors mounted directly above the tip of the water bottle sipper tube. Activity bouts were summed in 10-min intervals and stored on computer.

### Fear Conditioning Protocol

The FC chamber was constructed of aluminum walls on three sides and by clear acrylic for the front door and ceiling (Med Associates, Inc., Fairfax, VT, United States; 30.5 × 24.1 × 21 cm interior). The chamber was housed in a sound attenuating cabinet (Med Associates, Inc., Fairfax, VT, United States; 55.9 × 55.9 × 40.6 cm interior). Foot shocks were delivered *via* a shock stimulator/scrambler through the floor grid which consisted of 36 electrically conductive round stainless steel rods (0.32 cm diameter, 0.50 cm between rods; Med Associates, Inc., Fairfax, VT, United States). Current flow (mA) through the rods was confirmed by a current stimulation test module (Med Associates, Inc., Fairfax, VT, United States). The floor of the chamber was modified by installing a white plastic (Delrin™, Dupont) floor (1/2″ thick) beneath the rods. Grooves matching the diameter of the rods were cut into the plastic block at a depth equal to the radius of the rods so that there were no gaps between the rods and plastic block. A speaker located near the ceiling of the cabinet delivered auditory tones (80 dB, 1,000 Hz). Tone parameters were confirmed by monitoring the emitted sound with a microphone placed 0.25 cm above the grid floor that was connected to an amplifier and analog-to-digital converter (Med Associates, Inc., Fairfax, VT, United States). Air turnover in the cabinet was provided by an axial ventilation fan. Illumination was provided by two low-voltage lights mounted on the ceiling. For cleaning, the white plastic block was removed and the block, grid floor, walls, and ceiling of the chamber were cleaned with 70% ethanol between animals. During testing for responses to the cued stimulus (i.e., tone), the interior environment of the chamber was further modified with flexible sheets of white polypropylene that covered the floor and with an additional sheet that created a curved wall around the sides and back of the chamber (Med Associates, Inc., Fairfax, VT, United States). In this configuration, the chamber was cleaned between animals with a lemon-scented sanitizing solution.

**FIGURE 1:**
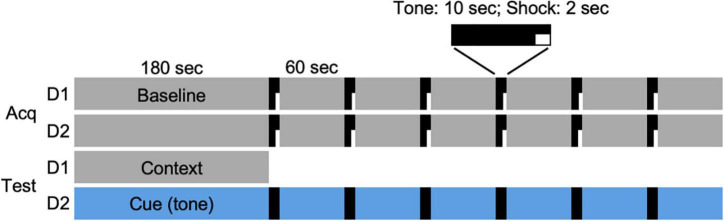
Fear conditioning protocol. Acquisition of conditioned responses was performed over 2 days (Acq D1, D2). On each day, a hamster was placed in the conditioning chamber and allowed 180 s to explore the chamber (Baseline). At the end of that time, a 10-s tone (80 dB, 1,000 Hz) was played that coterminated with a 2-s footshock (0.7 mA), followed by a 60-s observation interval. The tone/shock-observation sequence was repeated 6 times on each of 2 days. On the following day (day 3; Test D1), animals were returned to the chamber and allowed to explore for 180 s. On the final day (day 4; Test D2), the chamber was modified (see section “Materials and Methods”) and hamsters were allowed to explore the novel environment for 180 s, followed by 6 tone/observation sequences. Each animal was returned to the chamber at two time points (days 9–10 and 41–42) after the DPS treatment and tested for context (Test D1) and cued (i.e., tone; Test D2) responses over two consecutive days.

On days 1 and 2 of the FC protocol (Figure 1), an individual hamster was placed in the chamber and allowed to freely explore for 3 min. At the end of the 3-min period, a 10-s tone was played that coterminated with 2 s of electric current (0.7 mA) passed through the grid floor. The tone-shock pairing was followed by a 60-s observation interval. This tone/shock-observation sequence was repeated 6 times (day 1), and then repeated 24 h later (day 2) for a total of 12 sequences (Figure 1, Acq). On the day after the acquisition of the conditioned responses, each animal was returned to the chamber for an observation period (180 s; day 3). On the following day (day 4), the interior of the chamber was modified for the cued condition. Animals were allowed to freely explore the modified chamber for 180 s, after which, a tone was played for 10 s followed by a 60-s observation period. This tone-observation sequence was repeated 6 times without shocks. The administration of shocks and tones was controlled remotely by computer (EthoVision XT9, Noldus, Leesburg, VA, United States). All FC procedures were performed late in the afternoon, within 4 h of dark onset in the animal room, such that each animal was placed in the chamber at intervals of 24 h on days 1–4 of the protocol and testing.

### Induction of Circadian Arrhythmia: The Disruptive Phase Shift Protocol

The DPS protocol was initiated 14 days after completion of FC. This was accomplished by turning on the room lights at night for 2 h, beginning 5 h after lights-off (i.e., a 2-h light pulse from 2300 to 0100 h; daily lights on and off at 0200 and 1800 h, respectively). On the next day, the LD cycle was phase delayed by 3 h so that dark onset occurred 3 h later than it did on the previous night (i.e., time of lights-off shifted from 1800 to 2100 h; lights-on shifted from 0200 to 0500 h). Animals remained in this 16:8 LD cycle for the remainder of the study.

### Recording and Scoring of Freezing Behavior

Behavior of the animals was recorded continuously by a camera (Euresys PICOLO U4 H.264; resolution: 640 × 480 pixels, 30 frames/s) that was mounted on the cabinet ceiling directly over the foot shock chamber. Digitized recordings were saved to a computer and analyzed by video tracking software (EthoVision XT9, Noldus, Leesburg, VA, United States). Freezing behavior was detected by tracking the center body point at a sampling rate of 7.49 times/s. To differentiate the animal from the floor of the arena, a static subtraction method was used. With this method, the software takes a reference image of the arena without an animal. Once an animal is placed in the chamber, it is detected by comparing the pixels of each frame to the reference image. A new reference image was taken after cleaning the arena and before placing the next animal in the chamber.

An important experimental consideration in the automated quantification of freezing behavior is the duration of individual episodes of freezing. While rarely reported, this parameter is not only necessary to calculate the total time spent freezing, but may also provide additional insights into fear behavior, as reported here. Studies that use video tracking, or movement of the shock chamber itself, set the minimum duration for inactivity to between 0.5–2.0 s in order for inactivity to qualify as a bout of freezing (Goosens et al., 2003; Anagnostaras et al., 2010; Burman et al., 2014; Shoji et al., 2014; Gruene et al., 2015; Amorim et al., 2019; Kim and Cho, 2020; Totty et al., 2021). Based on our observations, we set a minimum bout duration of 1.0 s because that was the shortest interval of time that we could visually detect the absence of locomotion. An interval of inactivity was defined as freezing if the animal remained still without alerting, rearing, or grooming, and without body turns or head movements.

### Experimental Considerations for Siberian Hamsters

Siberian hamsters present unique challenges to successful FC not encountered in mice or rats. These animals have fine hair on the surface of their paws that makes it difficult for them to grip the stainless steel rods of the arena floor. During locomotion, their legs frequently slipped down between the rods. For this reason, we modified the floor with a plastic block placed underneath the rods into which grooves were cut to match the rod diameter (described above). While this allowed the hamsters to move about normally, it meant that all four paws may not be in contact with the rods during delivery of the shocks, and that the fine hair may attenuate electrical conduction, thus diminishing perceived shock intensity. Therefore, rather than rely on shock parameters used for mice, we performed a pilot study to find the optimal shock intensity and duration for Siberian hamsters (see section “Results”).

An additional challenge with Siberian hamsters is their lack of uniform coloration. For mice and rats, EthoVision (Noldus, Leesburg, VA, United States) provides a measure of “immobility” in which the system defines the image of an animal by the number of pixels occupied by its image. With this method, freezing is defined by the lack of changes in these pixels from video frame to frame. While this works well to define freezing in animals with uniform coloration, it did not work for Siberian hamsters because their dorsum is a mix of black, white, and gray stripes. For this reason, we used center point body tracking. Occasionally, this method would mistake the white floor for the animal, or fail to locate an animal when it was in a corner of the arena or if it made quick ambulatory movements. While such errors were rare (< 5% of frames), we were able to correct them by manually relocating the red tracking dot in each frame where the error occurred.

### Experimental Protocol and Data Analysis

The FC protocol was administered to male and female hamsters that were 2–3 months of age. The conditioning procedure was carried out over 2 days (Figure 1; Acq D1, D2). Animals were then tested for memory of contextual and cued associations on the following 2 days (Figure 1; Test D1, D2). Fourteen days later, the DPS protocol was administered. Animals were then tested twice more, on days 9–10 and 41–42 after the DPS treatment. A group of entrained age-matched control animals was administered the FC protocol and tested at the same time points but were not exposed to the DPS protocol.

The presence or absence of circadian locomotor rhythms was determined by chi-square periodogram analysis (ClockLab, Actimetrics, Wilmette, IL, United States) with the significance level set at *P* < 0.01. Freezing behavior was quantified by the percentage of time spent freezing, the number of freezing episodes (i.e., bouts), and by the duration of those individual bouts. Statistical comparisons of these measures were made between groups by ANOVA and with repeated measures for tests of context recall over time (i.e., BL, Pre-DPS, D9, D41). Freezing behavior in the cued (i.e., tone) conditions was also evaluated by ANOVA between groups and with repeated measures for presentation of the 6 tones. Dunnett’s correction was applied for all *post hoc* pairwise comparisons of context and tone compared to baseline (BL) values. Data are presented as mean ± SEM. Statistical comparisons are indicated in figures by asterisks (*) for between group comparisons and by hashtags (#) when data from groups were combined.

## Results

### Determination of Optimal Shock Intensity and Duration

**FIGURE 2:**
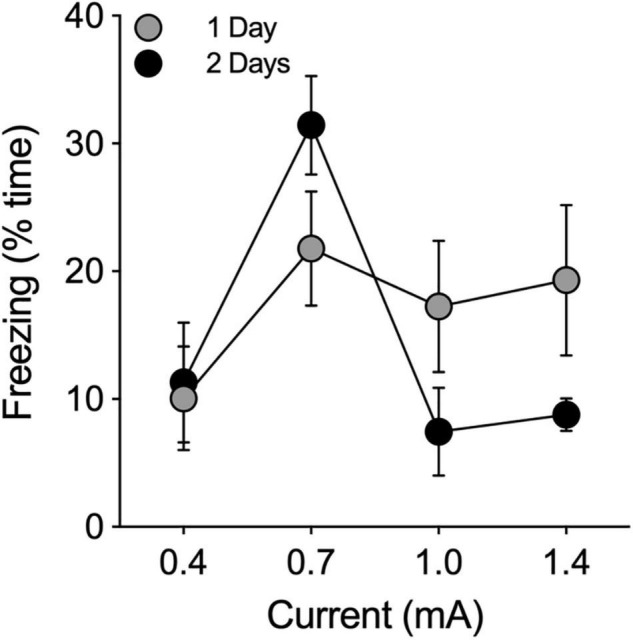
Pilot study to determine optimal shock intensity. Footshocks (2 s) were administered 6 times, separated by intervals of 60 s, on a single day (1 Day; filled gray circles) or on two consecutive days (2 Days; filled black circles) for a total of 6 or 12 shocks, respectively. Animals were returned to the chamber 24 h later and evaluated for freezing behavior. Freezing is expressed as the percentage of time the animal froze in 180 s. Optimal results were obtained at 0.7 mA.

Shock intensities of 0.4, 0.7, 1.0, and 1.4 mA were administered at durations of 0.5, 1.0, and 2.0 s to 12 separate groups of male hamsters (*n* = 3/group) to determine the optimal shock intensity and duration (*n* = 36 total). For each tone/shock combination, animals were allowed to explore the chamber for 180 s during a baseline (BL) period that was followed by 3 tone-shock pairings, each of which was followed by an observation period of 60 s. Twenty-four hours after shock administration, animals were returned to the chamber and observed again for freezing behavior. None of the shock combinations increased freezing behavior above BL levels. Therefore, we ran the experiment again with new groups of animals at the four shock intensities with 6 tone-shock pairings at a shock duration of 2 s (*n* = 12). This produced only a modest increase in freezing above baseline levels (Figure 2). We then tested 4 groups of animals (*n* = 3 each) at the same four different intensity levels using a shock duration of 2 s. These 4 groups were administered 6 tone-shock pairings on each of two consecutive days (i.e., 12 tone/shock pairings; Figure 2). There was no effect at the lowest shock intensity (0.4 mA). At the highest intensity (1.4 mA), animals responded by increasing their locomotor activity rather than freezing. Maximum levels of freezing were obtained at a shock intensity of 0.7 mA (2 s) administered 6 times each day for 2 days (i.e., 12 tone-shock pairings; Figure 2). For cued conditioning, we paired the foot shocks with a tone of 80 dB (1000 Hz, 10 s) that coterminated with each shock (2 s). This tone produced satisfactory results, so we did not perform experiments to further optimize the decibel level and frequency. Application of this protocol to a group of female hamsters (*n* = 6) produced results that were comparable to males (data not shown).

### Acquisition of Conditioned Fear

The FC protocol was administered to 60 hamsters. During the baseline (BL) recording on day 1 of the protocol, 12 animals appeared to exhibit neophobic behavior in the conditioning chamber. This behavior was manifested as abnormally high levels of freezing (> 65% time) during BL, followed by rapid increases in locomotor activity and decreased freezing in the observation intervals following the footshocks. An additional 8 animals did not maintain circadian arrhythmia for a sufficient number of days after the DPS protocol to assign them to any of the experimental groups. In total, these 20 animals were excluded from the study.

**FIGURE 3:**
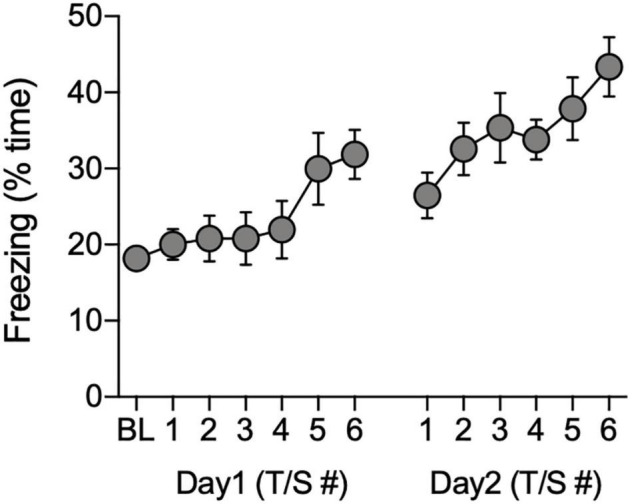
Total time freezing during acquisition of conditioned associations. Data show the percentage of time animals (*n* = 40) spent freezing during the baseline (BL) period and during the 60-s observation intervals that followed each tone/shock pairing (T/S) administered over two consecutive days.

Freezing behavior of the remaining 40 animals (*n* = 21 males, *n* = 19 females) gradually increased during the 60-s post-shock intervals during the acquisition phase of the FC task [*F*_(12, 468)_ = 4.28, *P* < 0.0001; Figure 3]. There were no significant effects of sex on freezing during acquisition (*P* > 0.05, ANOVA with repeated measures for tone-shock), therefore, the data were combined across sex (Figure 3).

### Sex Differences

In all statistical tests that follow, we examined the data for sex differences within groups, but none were found (all tests: *P* > 0.05). Therefore, data from males and females were combined. For the sake of clarity, the results of those individual negative tests are not reported.

### Determination of Circadian Arrhythmia

**FIGURE 4:**
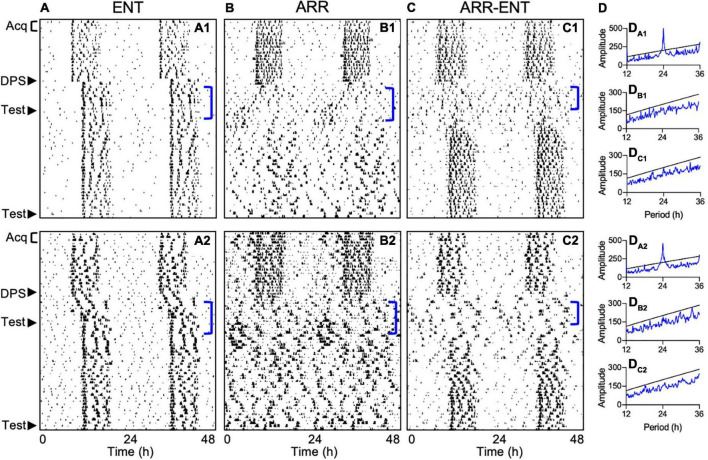
Representative actograms depicting different circadian responses to the DPS protocol. Locomotor activity is double-plotted over 48 h with successive days shown from top to bottom. Animals either reentrained to the LD cycle (ENT; **A1,A2**), became arrhythmic (ARR; **B1,B2**), or became ARR temporarily and then reentrained (ARR-ENT; **C1,C2**). Each phase of the study is indicated on the left of the actograms by Acq (Pre-DPS acquisition and testing), DPS (induction of arrhythmia), and Test (context and cue). Corresponding periodogram analyses for days 4–10 after the DPS treatment are shown to the right of the actograms **(D_A1_–D_C2_)**. Significant periodicities in the data are given by peaks (blue lines) above the level of significance (black line; *P* = 0.01). Periodogram analyses for days 31–42 are not illustrated.

Periodogram analyses were conducted to test for circadian arrhythmia at two time points during the study. The first one was limited to a 7-day period following the DPS treatment (days 4–10 post-DPS; Figure 4D). This limitation was necessary because the earliest onset of arrhythmia among ARR-ENT animals occurred 3 days after the DPS treatment with rhythms reemerging as soon as day 10 post-DPS. Therefore, we limited the periodogram analyses to 7 days (days 4–10 post-DPS) across animals in all 3 groups. The second time point for periodogram analyses was during the final 10 days of the experiment (days 33–42 post-DPS).

Fourteen days post-DPS treatment, animals were classified as either reentrained (ENT, *n* = 8; Figure 4A), arrhythmic (ARR, *n* = 12; Figure 4B), or temporarily arrhythmic for a minimum of 7 days before reentrainment to the LD cycle (ARR-ENT, *n* = 12, Figure 4C). A control group (*n* = 8) that was matched for age and sex to the ENT group, but not exposed to the DPS protocol, was tested in parallel to the three groups of experimental animals. We found no significant differences in any of the memory tests between this control group and the ENT experimental group, therefore, data from these two control groups were combined (ENT, *n* = 16) for all subsequent analyses.

### Contextual Fear Responses

**FIGURE 5:**
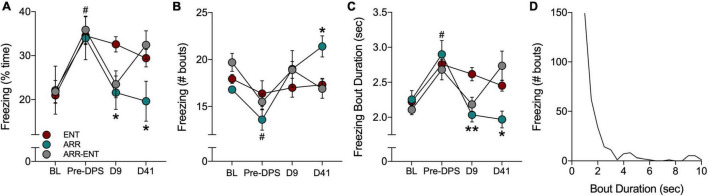
Freezing behavior during tests for contextual fear. Fear behavior was quantified by the total time spent freezing **(A)**, the number of freezing bouts **(B)**, and the duration of individual bouts of freezing **(C)**. Freezing was quantified during a baseline (BL) period and after the FC protocol (Pre-DPS), and again on days 9 and 41 after the DPS protocol. A frequency histogram of freezing bout durations for all bouts shows that most bouts are < 3.5 s **(D)**. Asterisks indicate significance for pairwise comparisons (ENT vs. ARR; ENT vs. ARR-ENT) with Dunnett’s correction applied (*P < 0.05, **P < 0.01). On day 9 (D9; panels **A,C**), asterisks indicate that both ARR and ARR-ENT groups differ significantly from the ENT group, whereas on day 41, asterisks only apply to the ENT vs. ARR comparison. Prior to the DPS treatment, all animals were entrained; there were no differences among them at the BL and Pre-DPS time points. Therefore, data were combined from all three groups for the BL vs. Pre-DPS comparison (^#^*P* < 0.0001).

There were no significant differences among ENT, ARR, and ARR-ENT groups in total time spent freezing, the number of freezing bouts, or in the duration of those bouts during: (1) the baseline observation period (BL; one-way ANOVA, *P* > 0.05 for all three measures), or (2) after administration of the FC protocol prior to the DPS treatment (Pre-DPS; one-way ANOVA, *P* > 0.05 for all three measures; Figure 5). Data from all three groups at these two time points were therefore combined. The combined data revealed a significant increase in time spent freezing [F_(1, 37)_ = 73.5, *P* < 0.0001; Figure 5A] and in bout duration [*F*_(1, 37)_ = 58.58, *P* < 0.0001; Figure 5C] from BL to Pre-DPS, whereas the number of freezing bouts decreased over this same interval [*F*_(1, 37)_ = 9.40, *P* = 0.005; Figure 5B].

Contextual memory was tested again 9 days after exposure to the DPS protocol. Animals that were circadian-arrhythmic at that time (i.e., ARR and ARR-ENT groups) exhibited levels of freezing behavior that were similar to values observed during BL (Figure 5). The total time spent freezing was significantly lower in ARR and ARR-ENT hamsters compared to the ENT group [one-way ANOVA; *F*_(2, 37)_ = 5.76, *P* = 0.008; *post hoc* comparisons: ARR, *P* = 0.025; ARR-ENT, *P* = 0.018; Figure 5A]. A similar decrease was observed for freezing bout duration in ARR and ARR-ENT hamsters compared to the ENT group [one-way ANOVA; *F*_(2, 37)_ = 8.10, *P* = 0.002; *post hoc* comparisons: ARR, *P* = 0.004; ARR-ENT, *P* = 0.007; Figure 5C], but not for the number of freezing bouts (one-way ANOVA, *P* > 0.05; Figure 5B).

The final test of contextual memory was performed on day 41. Among the ENT hamsters, total time spent freezing, the number of bouts, and bout duration did not differ significantly from day 9 values (repeated measures *t*-tests, *P* > 0.05 for all comparisons; Figures 5A–C). Likewise, among ARR animals, the values for these three measures did not change significantly from day 9 to day 41 (repeated measures *t*-tests, *P* > 0.05 for all comparisons). ARR animals maintained levels of freezing behavior on day 41 that were significantly different from those obtained in the ENT group for total time spent freezing [one-way ANOVA; *F*_(2, 37)_ = 3.35, *P* = 0.039; *post hoc* test, ARR vs. ENT, *P* = 0.033; Figure 5A], number of bouts [one-way ANOVA; *F*_(2, 37)_ = 4.68, *P* = 0.018; *post hoc* test, ARR vs. ENT, *P* = 0.014; Figure 5B], and bout duration [one-way ANOVA; *F*_(2, 37)_ = 4.19, *P* = 0.025; *post hoc* test, ARR vs. ENT, *P* = 0.013; Figure 5C].

Reentrainment of rhythms in the ARR-ENT group restored levels of freezing behavior on day 41 that were comparable to those observed prior to the induction of arrhythmia (i.e., Pre-DPS; Figures 5A–C). There were no significant differences between ARR-ENT and ENT groups on day 41 in the total time spent freezing, number of bouts, and bout duration (*P* > 0.05 for all comparisons; Figure 5).

We constructed a frequency distribution of the freezing bouts observed in this study to better understand fear behavior in Siberian hamsters. The shape of the histogram shows that most bouts were less than 3.5 s in duration (Figure 5D), with longer bouts occurring occasionally. For clarity’s sake, we set the upper limit at 10 s. Although rare, we did observe bouts of freezing that lasted longer than 20 s.

### Cued Fear Responses

**FIGURE 6:**
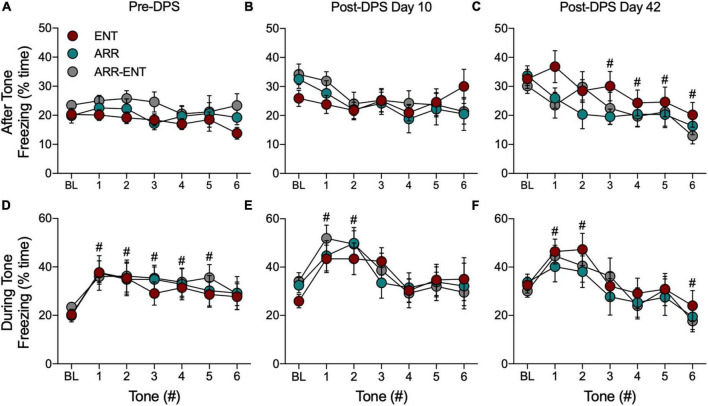
Freezing behavior during tests for cued fear responses. For these data, BL refers to the 180-s interval in the modified chamber that precedes presentation of the tones (Figure 1, Test D2). The total time spent freezing during BL and during the 60-s intervals after each tone before the DPS treatment (Pre-DPS; **A**), on day 10 **(B)**, and on day 42 **(C)** after the treatment (Post-DPS). Time spent freezing during the tones is given for the same time points **(D–F)**. Data are presented from each group, but because there were no significant group differences, data were combined for statistical analyses (^#^indicates 0.0001 < *P* < 0.05 compared to BL). Please note that the BL data are the same data for both conditions (i.e., after, during); it has been plotted for both conditions to facilitate comparison to the subsequent tone responses.

The percent time freezing was quantified for the 10-s intervals during presentation of each tone and for the 60-s intervals that followed the tone. For cued responses, baseline (BL) refers to the 180-s period in the modified chamber environment that preceded the presentation of the tones (i.e., Figure 1, Test D2). Unlike our findings for contextual fear, circadian condition (i.e., ENT, ARR, ARR-ENT) did not predict the amount of freezing behavior elicited in the cued (i.e., tone) condition. Analyses of data combined from all three groups were therefore limited to total time spent freezing to determine whether hamsters responded to the tones. For each panel in Figure 6, we performed a two-way ANOVA with repeated measures for tone. We did not find a significant effect of circadian rhythm condition on the total time spent freezing after or during the tones at any phase of the study (*P* > 0.05 for circadian condition).

Because we found no differences among ENT, ARR, and ARR-ENT groups, cued response data were combined across these groups for all subsequent comparisons. Freezing during presentation of the tones rapidly increased and then gradually declined during subsequent tones (Figures 6D–F). There were significant main effects of the tones for time spent freezing during the pre-DPS phase of the study [*F*_(6, 273)_ = 3.17, *P* = 0.005; Figure 6D], and on days 10 [*F*_(6, 273)_ = 4.49, *P* = 0.0002; Figure 6E], and 42 [*F*_(6, 273)_ = 6.38, *P* < 0.0001; Figure 6F]. *Post hoc* pairwise comparisons revealed several instances where freezing behavior differed significantly from BL values (# 0.0001 < *P* < 0.05 compared to BL).

For the 60-s observation intervals after the tones, there was no main effect for tone (one-way ANOVA, *P* > 0.05) prior to the DPS treatment (Figure 6A) or on day 10 after the DPS treatment (one-way ANOVA, *P* > 0.05; Figure 6B). By day 42, however, freezing behavior continued to decrease significantly after each tone [*F*_(6, 273)_ = 3.82, *P* = 0.001; Figure 6C].

Each time animals were tested for cued responses (Pre-DPS, D10, D42), a BL period of observation preceded presentation of the tones. We compared freezing behavior across BL periods and found that the total time spent freezing during these periods increased significantly after the DPS protocol [*F*_(2, 117)_ = 15.73, *P* < 0.0001; for both *post hoc* comparisons, Pre-DPS vs. D10 and Pre-DPS vs. D42; Figure 6].

## Discussion

As with mice and rats, Siberian hamsters learned to associate an aversive event with the context in which it occurred and increased freezing in response to an auditory cue that preceded the event. The impact of circadian arrhythmia on memory recall for context was substantial; loss of circadian timing reduced freezing to baseline levels despite exposure to 12 footshocks that were spread over 2 days. Upon spontaneously recovering entrained rhythms, hamsters exposed to the shock chamber expressed high levels of freezing. Circadian arrhythmia did not, however, influence freezing in the cued condition. Responses to the tones were similar among entrained and arrhythmic hamsters. Unlike mice and rats, Siberian hamsters did not exhibit increased freezing in the 60-s intervals after the tones, but did increase freezing during the tones. Fear behavior rose sharply during the first few tones and then declined thereafter, thus indicating that hamsters had acquired the tone/shock association. These data demonstrate that circadian arrhythmia, arising from an arrhythmic, but genetically and neurologically intact SCN, can suppress recall of an aversive memory.

Exposure to the FC protocol greatly reduced the number of animals that typically become permanently arrhythmic after the DPS treatment. In past studies, 40–60% of hamsters in any cohort remain arrhythmic (Ruby et al., 2008; Fernandez et al., 2014), but in the present study, only 20% did so when the DPS protocol was administered 14 days after FC. In a prior effort, we administered the DPS treatment 3 days after FC and found that only 1 animal out of 30 became arrhythmic (Ruby, unpublished observations). This reduction in efficacy may be due to reduced photosensitivity in the circadian system that can result from FC procedures (Amir and Stewart, 1998; Funk and Amir, 1999), and which might have diminished the phase-shifting effects of the light signals used in the DPS protocol. This effect might have also impacted animals that were deemed ARR. For example, one can see some residual rhythmicity in some ARR animals immediately after the DPS treatment and near the end of the experiment (Figures 4B1,B2). These transient periods of rhythmic activity were insufficient, however, to produce significant peaks in the periodograms when evaluated by either the Chi-square periodogram or Lomb-Scargle methods (data not shown), and were also insufficient to improve memory. These results should not be surprising though, as even modest disruptions in entrainment can impair memory (Ruby et al., 2015).

In rodents, freezing is characterized by stereotypical postures accompanied by a lack of movement, except for respiration (Blanchard et al., 1990; Eilam, 2005). The challenge of differentiating such postures from simple immobility by automated scoring methods, such as video recording or chamber displacement, has led investigators to redefine freezing as the absence of all non-respiratory related movement (Goosens et al., 2003; Anagnostaras et al., 2010; Burman et al., 2014; Shoji et al., 2014; Gruene et al., 2015; Amorim et al., 2019; Kim and Cho, 2020; Totty et al., 2021). Other manifestations of fear, such as fleeing or avoidance cannot be quantified due to the physical constraints of shock chambers. This constraint may be overcome, however, by using methods that are more ecologically valid, such as the shock-probe test that allows animals to flee and avoid an aversive stimulus in addition to freezing (Lehmann et al., 2006). Such tests require more time and effort, but they produce more meaningful data than can be obtained with a shock chamber because they allow animals to express a greater range of species-relevant fear behaviors.

We used freezing bout duration as a metric of fear to better understand how Siberian hamsters express fear in a laboratory setting. Hamsters did not freeze more often as fear increased, but instead, maintained their immobile state for longer periods. In the wild, freezing is a continuous state of threat assessment, the duration of which is determined by continuous monitoring of predators and by deciding when to switch among various defensive behaviors (Blanchard et al., 1990; Eilam, 2005). The strategy of prolonging freezing bouts might be more characteristic of burrow dwelling animals, such as Siberian hamsters, that use sustained periods of immobility to avoid detection by predators on the surface above them (Blanchard et al., 1990). Thus, in addition to activation of the freezing response, maintenance of the freezing state might be an ethologically relevant behavior that can also be conditioned in the laboratory. Whether mice and rats increase bout duration similarly to hamsters is unknown, but such a comparison would produce ethologically valuable data.

Unlike mice and rats, hamsters did not increase freezing behavior during the period following the tone in the cued condition, but did so only during tone presentation. While this could mean that hamsters formed a weak association to the cue, we consider this unlikely because the first tone elicited consistent increases in freezing at each phase of the study. This difference might be better explained by habituation. The first cued session elicited a sharp increase in freezing followed by a gradual decline, with declines occurring more rapidly on days 10 and 42. Alternatively, hamsters might differ from mice and rats in that fear responses do not carry over to the observation period, but instead require the direct presence of a perceived threat or its predictive cues to elicit freezing.

The differential effects of circadian arrhythmia on contextual and cued fear responses might be explained by the neural circuitry linking the SCN to fear expression circuits. Historically, memory related to contextual and cued FC was segregated to circuits within the hippocampus and amygdala, respectively. A current understanding of these neural substrates emphasizes the functional specificity of their diverse subregions as well as their interconnectedness in eliciting and suppressing fear behaviors (Izqueirdo et al., 2016; Yang and Wang, 2017; Asok et al., 2019). Nevertheless, there is a growing consensus that the lateral amygdala remains the primary region for conditioned responses to auditory cues while the hippocampus is critical for consolidating contextual information in long-term memory (Izqueirdo et al., 2016; Yang and Wang, 2017). Thus, circadian arrhythmia appears to have a greater impact on memory processes that depend on the hippocampus than those that depend on the amygdala.

Within the hippocampus, the dentate gyrus has received attention for its role in contextual FC. The dentate-CA3 microcircuit mediates encoding and discrimination of contextual information (Besnard et al., 2019, 2020; Rizzi-Wise and Wang, 2021). Optogenetic studies have shown more directly that the dentate mediates the recall of contextual fear memories (Liu et al., 2012; Ramirez et al., 2014). In our prior research, we found that circadian arrhythmia increased synaptic inhibition and attenuated cholinergic signaling in dentate granule cells (McMartin et al., 2021). Arrhythmia also disrupted theta oscillations, which are necessary for successful hippocampal encoding of objects and events (Loewke et al., 2020). Spatial memory can, however, be rescued in arrhythmic hamsters by several weeks of daily scheduled feeding (Ruby et al., 2017), which suggests that scheduled feeding can restore the normal balance of excitation/inhibition in the dentate. While the effects of SCN arrhythmia on dentate function and theta oscillations are sufficient to account for the memory deficits reported here, we cannot rule out the possibility that functional deficits in the amygdala contributed to the contextual memory impairments (Hobin et al., 2003; Izqueirdo et al., 2016; Yang and Wang, 2017).

In addition to the dentate gyrus, recent studies have established diverse roles for the various subregions of the lateral septum (LS) in conditioned fear behavior, and for the ventral lateral septum (LSv) in the suppression of freezing behavior. The LSv should be of interest to chronobiologists because it is the sole region to which the SCN projects within the limbic system, with reciprocal connections to the SCN and other nuclei in the anterior hypothalamus (reviewed in Ruby, 2021). Mongeau et al. (2003) performed an unbiased histological analysis of *c-fos* mRNA expression in over 70 brain regions from mice that exhibited freezing behavior in response to an aversive stimulus. Of all brain regions analyzed, the greatest density of cells expressing *c-fos* was in the LSv. Furthermore, optogenetic stimulation of CA3 terminals in the LSv suppressed freezing in a context previously associated with footshocks (Besnard et al., 2020; Rizzi-Wise and Wang, 2021). Thus, inputs to the LSv, such as those from the SCN, are anatomically positioned to suppress freezing behavior that would normally be elicited by contextual conditioning.

## Conclusion

The FC work presented here extends our earlier work with Siberian hamsters on spatial and recognition memory tests to show that arrhythmia in the SCN produces a distinct cognitive phenotype. This phenotype is characterized by binary responses to memory tasks that rely on hippocampal processing. For spatial and recognition memory, the task scores of arrhythmic hamsters are not simply lower than control animals. Rather, they fail completely, never performing better than chance (reviewed in Ruby, 2021). Likewise, in tests of contextual fear, arrhythmia did not just reduce freezing by a few percent, but instead, suppressed it back down to baseline levels. These findings stand in stark contrast to animals rendered circadian-arrhythmic by SCN lesions which exhibit little to no memory impairments (Ruby, 2021). The differing effects of these two models of arrhythmia on memory have important implications for understanding cognitive impairment in humans. Circadian dysfunction accelerates the onset of dementia and Alzheimer’s disease among aged populations (Tranah et al., 2011; Covell et al., 2012; Stranahan, 2012; Coogan et al., 2013), though the SCN remains a resilient structure that exhibits very little atrophy in older individuals (Fernandez et al., 2021). Thus, our studies suggest that age-related memory impairments are likely the result of active interference by a malfunctioning SCN, and not the result of SCN cell loss. Future studies aimed at improving cognitive outcomes *via* the circadian system will need to incorporate this dynamic into potential therapies.

## Data Availability Statement

The raw data supporting the conclusions of this article will be made available by the authors, without undue reservation.

## Ethics Statement

The animal study was reviewed and approved by the Stanford University’s Administrative Panel on Laboratory Animal Care (Animal Use Protocol #14988).

## Author Contributions

AS, F-XF, and NR: conceptualization and methodology. AS and NR: formal analysis and data curation. AS and JF: investigation. HH and NR: resources. NR: writing—original draft preparation, supervision, and funding acquisition, and supervision. F-XF and NR: writing. AS, F-XF, and HH: review and editing. All authors contributed to the article and approved the submitted version.

## Conflict of Interest

The authors declare that the research was conducted in the absence of any commercial or financial relationships that could be construed as a potential conflict of interest.

## Publisher’s Note

All claims expressed in this article are solely those of the authors and do not necessarily represent those of their affiliated organizations, or those of the publisher, the editors and the reviewers. Any product that may be evaluated in this article, or claim that may be made by its manufacturer, is not guaranteed or endorsed by the publisher.
